# Clinical Algorithm‐Guided Approach to Botulinum Toxin Type A Treatment for Axial Postural Abnormalities in Parkinson's Disease

**DOI:** 10.1002/mdc3.70408

**Published:** 2025-11-05

**Authors:** Giacomo Argenziano, Giovanna Squintani, Serena Camozzi, Ilaria Di Vico, Marialuisa Gandolfi, Carlo Alberto Artusi, Michele Tinazzi, Christian Geroin

**Affiliations:** ^1^ Neurology Unit, Neuromotor and Rehabilitation Department Azienda USL‐IRCCS Reggio Emilia Reggio Emilia Italy; ^2^ Neurology and Neurophysiology Unit, Azienda Ospedaliera Universitaria Integrata Verona Italy; ^3^ Neurology Unit, Movement Disorders Division, Department of Neurosciences, Biomedicine and Movement Sciences University of Verona Verona Italy; ^4^ Department of Neurosciences, Biomedicine and Movement Sciences University of Verona Verona Italy; ^5^ Department of Neuroscience “Rita Levi Montalcini” University of Turin Turin Italy; ^6^ Neurology 2 Unit, A.O.U. Città della Salute e della Scienza di Torino Torino Italy; ^7^ Department of Surgery, Dentistry, Paediatrics and Gynaecology University of Verona Verona Italy

**Keywords:** botulinum toxin, axial postural abnormalities, Pisa syndrome, camptocormia, EMG polygraphy

## Abstract

**Background:**

Axial postural abnormalities (APAs) are common, disabling complications of Parkinson's disease (PD) with unclear pathophysiology. The presence of hyperactivity in multiple trunk muscles supports the use of botulinum toxin (BTA). However, its application is complex, due to the lack of standardized clinical and electrophysiological approach.

**Objectives:**

To evaluate BTA injections effectiveness using a clinical algorithm that integrates trunk angle severity and electromyography (EMG)‐detected muscle hyperactivity in patients with APAs.

**Methods:**

This is prospective, open‐label, pilot study. A novel decisional algorithm guided muscle selection, based on the bending degree and EMG findings. PD patients with different APAs underwent trunk angle measurement and EMG polygraphy of axial muscles in the standing position and during trunk activation. Primary outcome was the change in trunk misalignment measured before and one month after BTA injection. Secondary outcomes were the Clinical Global Impression of Change‐Improvement (CGI‐pain and CGI‐posture) and the Numeric Rating Scale (NRS) for pain.

**Results:**

Among 52 PD patients, 20(38.5%) were eligible for treatment. At one‐month follow‐up, lateral trunk flexion (LTF) improved [LTF angle from 11.5 (CI 7.1–15.9) to 9.9, (CI 5.2–14.7) *P* = 0.013], while anterior trunk flexion (ATF) did not (thoracic fulcrum ATF from 41.4 to 40.6, *P* > 0.05; lumbar fulcrum ATF from 25.7 to 24.5, *P* > 0.05). CGI‐posture improved in 45% of cases, CGI‐pain improved in 50% of cases, and NRS decreased from 6.3 to 4.8 (*P* = 0.010).

**Conclusions:**

A clinical and neurophysiological algorithm integrating measures of trunk bending and EMG‐detected muscle hyperactivity can optimize BTA treatment for APAs in PD, improving motor/ non‐motor outcomes.

Axial postural abnormalities (APAs) are debilitating motor complications of Parkinson's disease (PD). They have been associated with increased disability and functional decline.^1^, ^2^ Moreover, APAs are less rare than expected, with an overall prevalence reported to exceed the 20% among patients with PD.^3^, ^4^ The most common are camptocormia (CC) and Pisa syndrome (PS), with a prevalence of approximately 10% and 8%, respectively.^3^, ^4^ CC is defined as a marked forward trunk flexion with a lumbar fulcrum (lf) >30° and/or thoracic fulcrum (uf) >45°. PS is defined as a lateral trunk flexion of >10°.^5^ APAs appear in the sitting or standing position, tend to worsen during walking, can be alleviated by passive mobilization or lying positioning^5^ and may negatively impact the patients’ quality of life. In fact, APAs are frequently associated with higher severity of motor symptoms, postural instability, leading to an increased risk of falling and back pain.^1^, ^6^, ^7^, ^8^ Considering the associated disability, the lack of response to dopaminergic therapy and the fact that their pathophysiology is largely unknown, APAs represent a therapeutic challenge.^7^, ^8^, ^9^

The pathophysiology of APAs involves both peripheral and central mechanisms.^7^, ^8^ The main hypothesis of a peripheral origin of APAs is based on detection of myopathic features in axial paraspinal muscles, that have been evidenced by inconsistent electromyography (EMG) findings (ie, small duration and amplitude, and polyphasic motor unit action potentials),^10^, ^11^ magnetic resonance imaging findings (MRI) (with either edema in early stage or muscular atrophy and fatty substitution in chronic phase),^8^, ^11^, ^12^, ^13^ and also confirmed by muscle biopsy studies in some patients with CC.^14^, ^15^ Myopathic changes on EMG have been hypothesized to become evident in later stages of the APAs, as part of a progressive muscle transformation that begins with edema and culminates in degeneration of the axial muscles.^6^, ^14^ Also, the observed myopathic changes did not allow for differentiation between a primary pathological process affecting axial paraspinal muscles (suggesting a causative role of myopathy) and a secondary consequence resulting from proprioceptive dysregulation of central origin, fixed posture, and subsequent muscle disuse.^16^, ^17^ No histological findings are available for PS, so muscular atrophy ipsilateral to bending, when observed, is probably secondary to muscular disuse, while contralateral atrophy may result from prolonged stretching stress.^12^, ^18^ In addition, degenerative spine disorders may be associated with APAs, which can have mechanical effects on bone and soft tissues, leading to painful compensatory postures.^7^, ^8^

Among the proposed mechanisms of central origin there is a hyperactivity of paraspinal and abdominal trunk muscles, whose origin was postulated to be dystonic. The hyperactivity of a given muscle was defined as high tonic activity during conditions that should be characterized by physiological EMG silence, such as at rest or during voluntary activity of antagonistic muscles.^11^, ^19^ Hyperactivity of axial muscles has been revealed by EMG both in CC^11^, ^20^, ^21^ and PS.^12^, ^18^, ^19^, ^22^, ^23^ The reported maneuvers that alleviate camptocormia in PD,^24^, ^25^ often referred as sensory tricks, support the hypothesis of dystonic origin. Other factors that are thought to contribute to the genesis of APAs are the basal ganglia dysfunction, proprioceptive and vestibular impairment of central origin. Visuospatial and cognitive deficits, along with drugs, such as dopamine agonists, have also been implicated.^2^, ^7^, ^8^, ^19^

The clinical management of CC and PS is challenging and requires a multidisciplinary approach. Pharmacological treatments, including adjustments in dopaminergic medications, gave equivocal results.^6^, ^8^, ^16^, ^26^ Physiotherapy, emphasizing postural correction and strengthening of the axial muscles, play a crucial role in the management strategy.^8^, ^16^, ^26^, ^27^, ^28^ Although dystonia as a driving factor for the establishment of APAs in PD are still debated, a considerable number of studies have used botulinum toxin (BTA) injection as a treatment option.^29^, ^30^, ^31^, ^32^, ^33^, ^34^, ^35^, ^36^, ^37^, ^38^, ^39^, ^40^, ^41^ They treated in most cases the muscles ipsilateral to the bending side according to the EMG pattern of hyperactivity found with polygraphic studies.^29^, ^33^, ^37^

However, the results of these studies were variable, and among APAs, PS showed the greatest improvements following BTA administration.^29^, ^30^, ^31^, ^32^, ^33^, ^34^, ^35^, ^36^, ^37^, ^38^, ^39^, ^40^, ^41^, ^42^ This inconsistency may stem from methodological differences in identifying target muscles for injection. Although previous studies^29^, ^30^, ^31^, ^32^, ^33^, ^37^, ^39^, ^40^, ^41^ have utilized EMG to detect hyperactive muscles, the limited benefits reported in some of these studies suggest that EMG alone may not be sufficient. Effective BTA infiltration requires both a precise assessment of trunk flexion severity and well‐reasoned prioritization of the APA to be treated, a need that becomes especially relevant when multiple APAs coexist.

Therefore, our study aimed to evaluate the effectiveness of BTA injections guided by a structured algorithm that combines trunk angle measurements with EMG evidence of muscle hyperactivity in PD patients with varying degrees of CC and PS. The novelty of this study lies in applying a systematic clinical and neurophysiological approach to guide BTA treatment of APAs in PD, using a specific, and predefined algorithm with potential clinical practice utility.

## Methods

### Subjects

We prospectively studied 52 consecutive patients with PD and APAs who were a mix of follow‐up cases and newly referred to the Movement Disorders Outpatient Clinic from September 2022 to October 2024, at Verona Hospital. The study was approved by the Local Ethics Committee (CE2399) and was conducted in accordance with the Declaration of Helsinki. Inclusion criteria were clinically established diagnosis of PD according to the Movement Disorder Society (MDS) diagnostic criteria^43^ confirmed by a movement disorders specialist, the presence of PS, CC, or their mild forms according to recent MDS Task Force Diagnostic Criteria,^5^ modified Hoehn and Yahr stage ≤4, and the possibility of patient to stand without assistance. Exclusion criteria were: patients with isolated antecollis, those with recent (<6 months) changes in dopamine agonist therapy, patients with active treatment with deep brain stimulation, the presence of moderate–severe tremor during ON‐medication state (item 3.17 of MDS‐UPDRS score ≥3), ongoing anticoagulant therapy, history of exposure to antipsychotics, valproate, and anticholinergics as drugs potentially inducing APAs, severe bone deformities or spinal cord pathologies. During a preliminary screening visit we collected the following demographic and clinical data: age, gender, disease duration (years), duration of APAs (months), PD‐related therapy, modified Hoehn & Yahr scale, Levodopa Equivalent Daily Dose (LEDD), and MDS‐UPDRS III during ON state with the usual therapy. LEDD was calculated using conversion factors reported by Schade and colleagues.^44^

### EMG Assessment and BTA Treatment

Each patient underwent an eight‐channel muscle EMG, ultrasound‐guided, consisting of intramuscular simultaneous recording of bilateral thoracic iliocostal muscle at T8‐9 level (TP), lumbar iliocostal muscle at L2 level (LP), external abdominal oblique muscle (EAO) and rectus abdominis muscle (RA). The EMG system used was a Keypoint system (Dantec, Skovlunde, Denmark). Electrodes consisted of 18 mm length monopolar needles (or coaxial needles if muscles were covered by a significant amount of adipose tissue) inserted bilaterally as previously described.^11^, ^23^ Recordings were made in orthostatism at rest and during activation in different positions: during maximal trunk extension (for patients with ATF) and maximal lateral flexion contralateral to the site of patient's bending (for patients with LTF) up to maximal range of motion, for at least 10 s. Dystonic hyperactivity for LTF was defined as paraspinal muscular activation present at rest, that persisted during voluntary contralateral trunk flexion, thus indicating the characteristic co‐contraction of agonist and antagonist muscles described in dystonic pattern.^11^, ^19^ Dystonic hyperactivity for ATF was defined as anterior wall muscle activation present at rest and persisting during trunk extension, resulting in co‐activation of antagonistic muscles.^11^ Since the amplitude of the EMG pattern can be considered a surrogate for force,^45^ we defined muscular hyperactivity as the presence of muscular activity with an average amplitude of at least 0.2 mV lasting for a minimum duration of 1 second within an epoch recording (Fig. S1). In normal physiological conditions, an amplification of 0.2 mV generally does not show muscle activity at rest or during activation of the antagonistic muscles.

**Figure 1:**
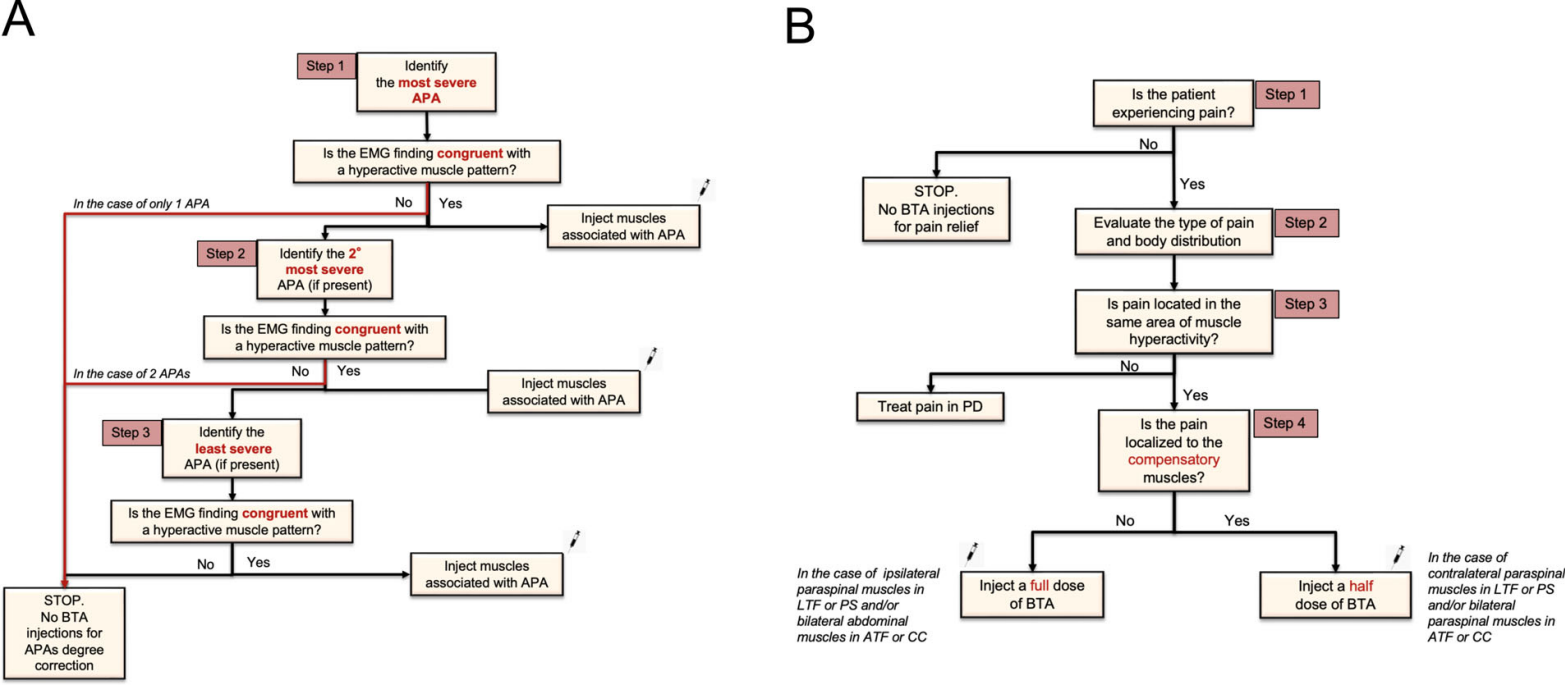
An illustration of the decision‐making algorithm used to appropriately select dystonic muscles as targets for BTA injection. This represents an expert opinion–based algorithm: botulinum toxin injection into hyperactive muscles should be cautiously considered on a case‐by‐case basis, guided by EMG findings, in carefully selected patients, under the care of experts.^8^ The algorithm is designed with two primary therapeutic goals: to improve the degree of trunk bending (Fig. 1, panel A) and/or to reduce pain (Fig. 1, panel B). The selection of muscles is guided by two main criteria: muscle hyperactivity, as assessed by EMG and the type and degree of trunk flexion, measured clinically using a software‐based method. Considering that patients may present with one or more APAs, ie, such as PS + CC with a thoracic fulcrum, the algorithm begins with the evaluation of the most severe condition (step 1)—defined as the APA associated with the highest degree of trunk bending. Once the most severe APA is measured and clinically identified, an EMG assessment is performed to evaluate the muscles potentially contributing to the abnormal posture. If EMG findings confirm muscle hyperactivity in these muscles, BTA is injected into hyperactive muscles. Conversely, if no hyperactivity is detected, the algorithm proceeds to assess the next (less severe) APA using the same evaluation process (step 2). This stepwise approach continues until hyperactive muscles are found and treated (step 3). Following the main aim of postural correction, the clinical focus shifts toward pain management. If the patient reports pain (step 1), it becomes important to identify the type of pain (step 2)—following the classification proposed by Mylius et al^53^—as well as its anatomical distribution. The next step involves determining whether the pain coincides with the site of muscle hyperactivity or not (step 3). If the pain does not correspond to a region of hyperactive muscle activity, it should be managed according to the recommendations for pain in PD.^52^ The final step (step 4) is to determine whether the pain is localized to compensatory muscles. If so, a half‐dose of BTA may be considered, for example, in the paraspinal muscles in CC or contralateral paraspinal muscles in PS. However, if the pain corresponds to hyperactivity ipsilateral to the bending side, as often seen in PS, a full dose of BTA may be appropriate.

For each patient, target muscles were identified after properly combining clinical and EMG data. Figure 1 represents the decision‐making algorithm applied for each patient (Fig. 1, panel A and B).

For injections, BTA (Abobotulinum toxin A) was used. The dilution employed was 500 IU in 1.25 mL of saline solution, with a total BTA dose ranging from 240 to 720 IU. Based on the extensive experience of the injecting neurophysiologist, data from the literature, and the tolerability profile of the injected BTA, we injected each muscle in two different sites, in order to increase the spread of botulinum toxin in treated muscles. BTA was performed under ultrasound guidance to confirm the correct injection site. The decision‐making algorithm was used to appropriately select dystonic muscles as targets for BTA injection. This algorithm was designed with two primary therapeutic goals: to improve the degree of trunk bending (Fig. 1, panel A) and/or to reduce pain (Fig. 1, panel B).

### APAs Assessment

Patients were evaluated before and 1 month after BTA treatment with primary and secondary outcomes. The primary endpoint was any change of angle in lateral or anterior trunk flexion after BTA injection. Measurement of the angle was performed using the tool AutoPosturePD.^5^, ^46^, ^47^, ^48^, ^49^ Secondary endpoints were self‐reported changes of posture and pain evaluated by the Clinical Global Impression of Change‐Improvement (CGI‐I) and any change in the Numerical Rating Scale (NRS) for pain after BTA injection. Douleur Neuropathique 4 questionnaires (DN4) was administered to assess the neuropathic features of pain.

### Statistical Analysis

Descriptive statistics included frequency tables for categorical variables and means and standard deviations for continuous variables. The Shapiro–Wilk test was used to verify whether the sample under examination had a Gaussian distribution. Parametric statistics (paired T test and Pearson correlations) were then used for variables with a normal distribution, while non‐parametric (Wilcoxon signed rank and Spearman correlations) tests were employed for variables with a non‐normal distribution. Statistical significance was set at *P* < 0.05. All analyses were performed using SPSS version 26 package.

## Results

Among 52 outpatients, five were excluded due to skeletal deformities: one had a fixed deformity since the age of 20 following a car accident, one had a fixed deformity associated with a severe resting tremor; the other three excluded patients presented fixed and severe spine deformities on the x‐rays. Of the remaining 47 patients, only 28 (59.6%) exhibited an EMG pattern consistent with dystonic hyperactivity, characterized by a mean amplitude of at least 0.2 mV. Patients who did not display dystonic hyperactivity did not report back pain and therefore were not proposed injections. Among the aforementioned 28 patients, 20 (71.4%) accepted the proposed BTA injections after providing written informed consent, while eight of them declined treatment for various reasons, including fear of injections and transportation difficulties which might have led to poor compliance with the scheduled assessment (see Table 1). The mean age of subjects was 74 years (standard deviation [SD] = 6 years). There was a slight predominance of males (*n* = 11; 55%) over females (*n* = 9; 45%). The mean duration of PD at the time of assessment was 9 years (SD = 4 years), and the mean duration of APAs was 26 months (SD = 14 months). MDS‐UPDRS part III mean score was 36 points (SD = 8 points), and the Modified H&Y mean score was 2.45 (SD 0.65). All subjects were under symptomatic dopaminergic therapy with Levodopa; 60% of subjects were regularly taking dopamine‐agonists (DA), while 25% of subjects were under Levodopa alone. Mean LEDD was 759.7 mg (SD 333.3 mg) (Table 1).

**TABLE 1:** Demographic and clinical data of patients with APAs

Patient	Age	Gender	PD Duration (Y)	APAs Duration (M)	MDS‐UPDRS III	mH&Y	DA	L‐Dopa alone	LEDD
1	59	F	4	24	18	1	Yes	No	331
2	73	M	10	48	30	2	No	Yes	600
3	80	M	5	48	43	4	No	Yes	575
4	84	M	12	36	47	3	No	No	550
5	80	F	14	48	33	3	Yes	No	656
6	72	M	8	36	49	2	Yes	No	1230
7	82	F	7	6	32	2	No	Yes	400
8	69	M	7	12	43	3	Yes	No	1335
9	69	M	4	36	33	1	No	Yes	800
10	78	M	5	24	31	2	No	Yes	700
11	76	M	16	24	32	3	Yes	No	1305
12	78	M	4	12	29	2	No	No	400
13	73	M	7	18	42	3	Yes	No	952
14	69	F	14	24	33	2	Yes	No	780
15	70	F	15	36	45	2	Yes	No	820
16	75	M	14	6	40	3	Yes	No	1356
17	75	F	14	36	32	3	Yes	No	965
18	79	F	4	24	36	2	No	No	400
19	63	F	9	12	25	2	Yes	No	610
20	67	F	9	12	43	2.5	Yes	No	430

*Abbreviations: APAs, axial postural abnormalities; DA, dopamine agonist; F, female; LEDD, Levodopa equivalent daily dose (mg); M, male; MDS‐UPDRS III, International Parkinson and Movement Disorder Society's revision of Unified Parkinson's Disease Rating Scale, item number 3; mH&Y, modified Hoehn & Yahr scale; M, Months; APA duration PD, Parkinson's disease; Y, years*.

Three out of 20 patients did not report pain. Seventeen patients experienced musculoskeletal pain and none reported neuropathic pain, as indicated by DN4 scores below the 4 points threshold (Table 2). The trunk bending angle values in the sagittal and coronal plane for each subject, prior to BTA treatment, are displayed in Table 2. In particular, 10 patients met the cut‐off values for uf‐ATF, nine met criteria for uf‐CC, 11 met the criteria for lf‐ATF, five patients met criteria for lf‐CC. Finally, 12 met criteria for LTF and six met criteria for PS, according to MDS cut‐off values.^5^ In the studied group, no patient exhibited an isolated APA. Table 2 also shows which muscles displayed EMG hyperactivity, which muscles were injected with BTA and the total injected dose. Most of the patients (75%) showed hyperactivity in the TP muscles, either bilaterally or ipsilaterally to the trunk bending site.

**TABLE 2:** Quantitative assessment of APAs severity (in degrees) and pain severity (NRS score) before BTA treatment; EMG‐detected dystonic hyperactivity from a polygraphic study, muscle treated with BTA; and total doses administered (in IU)

Patient number	uf‐ATF or CC	lf‐ATF or CC	LTF or PS	Side of trunk bending	NRS pre	DN4	EMG‐detected muscle hyperactivity	Muscles treated	Abo‐BTA total dose IU
1	36.4	19.7	3.4	L	8	0	Bilateral EO	Bilateral EO	480
2	46.4	38.9	5.2	R	0	0	Bilateral RA	Bilateral RA	480
3	56.8	25.2	6.7	R	7	0	Right TP and LP°	Right TP, LP	480
4	46.8	27.2	6.0	R	5	0	Bilateral TP and LP°	Right TP, LP	480
5	42.6	28.1	6.1	L	7	0	Left TP°	Left TP	240
6	49.9	61.4	27.1	R	8	0	Right TP°	Right TP	240
7	41.2	13.6	7.6	R	5	0	Bilateral EO	Bilateral EO	480
8	50.4	12.7	6.0	L	0	0	Bilateral TP°	Left TP	240
9	36.3	32.9	6.1	R	8	0	Bilateral TP (right prevalence°)	Right TP	240
10	48.3	24.1	2.8	R	7	0	Bilateral RA	Bilateral RA	480
11	51.3	26.5	28.9	L	8	0	Bilateral TP (left prevalence)	Left TP	240
12	36.2	25.0	5.6	L	9	0	Bilateral RA	Bilateral RA	480
13	56.8	26.0	6.1	R	5	0	Bilateral TP (right prevalence°)	Right TP	240
14	33.3	27.0	15.8	L	8	0	Left TP°	Left TP	240
15	15.6	32.1	6.8	L	8	0	Bilateral TP (left prevalence°)	Left TP	240
16	25.7	11.3	8.1	R	9	0	Bilateral TP°	Right TP	240
17	35.6	38.3	32.6	R	8	0	Bilateral TP and LP°	Right TP, LP	480
18	46.4	13.4	6.2	R	0	0	Bilateral TP°, right EO	Right TP, right EO	480
19	41.8	15.1	20.7	R	8	0	Bilateral TP	Right TP	240
20	31.3	16.1	22.3	R	9	0	Right EO	Right EO	240

*Note:* In these cases, according to the decision‐making algorithm, the first two APAs did not exhibit any muscle hyperactivity on EMG evaluation and were therefore not treated. Treatment with BTA was administered only in the final APA, where hyperactive muscles were identified. Importantly, we did not treat trunk bending patterns that were classified as normal postures based on the diagnostic criteria established by the MDS (Tinazzi et al, 2022).

Abbreviations: Abo‐BTA, abobotulinum toxin type A; DN4, Douleur Neuropathique 4 questionnaire; EO, external oblique muscle; lf‐ATF, lower fulcrum anterior trunk flexion; LP, lumbar paraspinal muscles; LTF, lateral trunk flexion; NRS, numeric rating scale for pain; RA, rectus abdominis muscle; TP, thoracic paraspinal muscles; uf‐ATF, upper fulcrum anterior trunk flexion.

### Efficacy of BTA Injection

**Figure 2:**
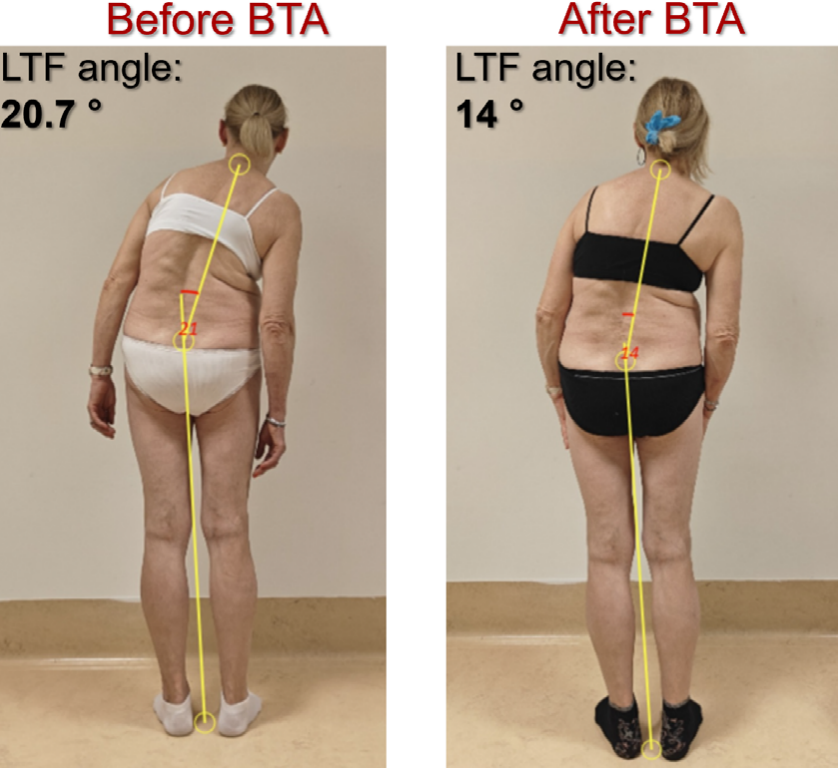
An improvement of right‐sided Pisa syndrome (PS) in patient n. 19 1 month after botulinum toxin (BT) injection. On the left hand side the lateral trunk flexion (LTF) angle value before treatment; on the right hand side the LTF angle value after BT, with an improvement of 6.7 degrees. BT, botulinum toxin; LTF, lateral trunk flexion.

One month after BTA injection we found a mild, statistically significative reduction of LTF angle [from 11.5° (CI: 7.1–15.9) to 9.9° (CI: 5.2–14.7), *P* = 0.013] and NRS [from 6.3 (CI: 5–7.8) to 4.8 (CI: 3.4–6.4), *P* = 0.010]. For the uf‐ATF and lf‐ATF angles degrees, we did not significantly change (Table 3, 4, 5, Fig. S2). An example of improvement of Pisa syndrome is reported in Figure 2.

**TABLE 3:** Quantitative assessment of APAs severity (in degrees) before and after treatment with BTA

P	uf‐ATF pre	uf‐ATF post	Δ uf‐ATF	lf‐ATF pre	lf‐ATF post	Δ lf‐ATF	LTF pre	Side	LTF post	Δ ‐LTF
1	36.4	36.4	0	19.7	16.3	−3.4	3.4	L	2.4	−1
2	46.4	45.7	−0.7	38.9	39.2	0.3	5.2	R	0.0	−5.2
3	56.8	53.4	−3.4	25.2	17.1	−8.1	6.7	R	20.7	14
4	46.8	48.4	1.6	27.2	26.2	−1	6.0	R	3.4	−2.6
5	42.6	38.5	−4.1	28.1	19.8	−8.3	6.1	L	3.3	−2.8
6	49.9	54.7	4.8	61.4	53.1	−8.3	27.1	R	24.6	−2.5
7	41.2	47.0	5.8	13.6	9.5	−4.1	7.6	R	9.3	1.7
8	50.4	46.0	−4.4	12.7	5.5	−7.2	6.0	L	1.0	−5
9	36.3	30.9	−5.4	32.9	25.3	−7.6	6.1	R	0.9	−5.2
10	48.3	36.4	−11.9	24.1	28.7	4.6	2.8	R	2.2	−0.6
11	51.3	52.4	1.1	26.5	27.5	1	28.9	L	25.3	−3.6
12	36.2	30.1	−6.1	25.0	31.0	6	5.6	L	4.0	−1.6
13	56.8	53.4	−3.4	26.0	27.5	1.5	6.1	R	0.6	−5.5
14	33.3	36.1	2.8	27.0	28.8	1.8	15.8	L	12.2	−3.6
15	15.6	25.3	9.7	32.1	39.3	7.2	6.8	L	7.5	0.7
16	25.7	27.4	1.7	11.3	15.5	4.2	8.1	R	7.4	−0.7
17	35.6	35.2	−0.4	38.3	32.9	−5.4	32.6	R	34.7	2.1
18	46.4	48.5	2.1	13.4	16.6	3.2	6.2	R	4.0	−2.2
19	41.8	36.4	−5.4	15.1	11.8	−3.3	20.7	R	14.0	−6.7
20	31.3	30.7	−0.6	16.1	18.7	2.6	22.3	R	21.7	−0.6

*Note:* The table also presents the delta values (difference between post‐treatment and pre‐treatment) of each of these variables.

Abbreviations: lf‐ATF, lower fulcrum anterior trunk flexion; LTF, lateral trunk flexion; uf‐ATF, upper fulcrum anterior trunk flexion.

**TABLE 4:** Quantitative assessment of and pain severity (NRS) before and after treatment with botulinum toxin. The table also presents the delta values (difference between post‐treatment and pre‐treatment) of NRS and the clinical global impression ‐ improvement (CGI‐I) scale for posture and pain for each subject

p	NRS pre	NRS post	Δ NRS
1	8	5	−3
2	0	0	0
3	7	0	−7
4	5	0	−5
5	7	4	−3
6	8	6	−2
7	5	7	2
8	0	0	0
9	8	7	−1
10	7	7	0
11	8	8	0
12	9	9	0
13	5	5	0
14	8	8	0
15	8	4	−4
16	9	5	−4
17	8	6	−2
18	0	0	0
19	8	7	−1
20	9	9	0

Abbreviation: NRS, numeric rating scale for pain.

**TABLE 5:** Change of APAs angle degrees and NRS at follow up

Angle	Mean trunk bending angle pre‐BTA injection	Mean trunk bending angle post‐BTA injection (1 month of follow‐up)	Statistics (*P < 0.05)
Uf‐ATF	41.4	40.6	0.468
Lf‐ATF	25.7	24.5	0.313
LTF	11.5	9.9	0.013*
NRS	6.3	4.8	0.010*

Abbreviations: BTA, botulinum toxin type A; lf‐ATF, lower fulcrum anterior trunk flexion; LTF, lateral trunk flexion NRS, numeric rating scale for pain; uf‐ATF, upper fulcrum anterior trunk flexion.

**Figure 3:**
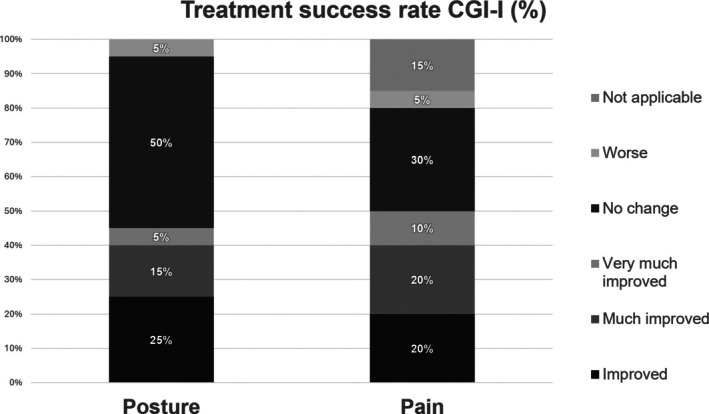
Treatment success rate (%) in posture and pain after BTA.

Regarding the CGI‐I score for posture, nine patients reported an improvement in posture (CGI‐I posture score ranging from 1 to 3), 10 patients showed no changes (CGI‐I posture score = 4), and one patient reported a worsening of posture (CGI‐I posture score = 5). Ten patients experienced a subjective reduction in pain (CGI‐I pain score ranging from 1 to 3), 6 reported no subjective change (CGI‐I pain = 4), 1 patient experienced a worsening of pain (CGI‐I pain score = 5); 3 patients had no pain associated with APA before treatment (Fig. 3). No significant side effects were reported, apart from a transient injection site discomfort. NRS score decreased from 6.3 to 4.8 (*P* = 0.010) (Table 5, Fig. S3). We did not find significant correlation between a reduction of NRS score and the decrease of the degree of APAs.

## Discussion

We evaluated the effect of BTA injection in patients with PD who exhibited different APAs. We demonstrated that BTA is safe, can ameliorate posture and alleviate pain related to APAs. To choose which muscle to infiltrate, we relied on the algorithm shown in Figure 1, which is based on either the most severe APA correlated to EMG pattern or the presence of painful muscles. Considering patients with LTF, the TP and the EAO ipsilateral to bending were the most frequently treated muscles, as they are, from a biomechanical perspective, the main responsible actors for LTF. In contrast, hyperactivity of the contralateral muscles appears to be a compensatory mechanism,^12^ which, in our opinion, should be preserved and ideally not weakened. The strategy of targeting paraspinal or anterior abdominal wall muscles ipsilateral to the side of LTF in order to contrast the APA, has been adopted by several authors in the management of PS in PD.^29^, ^31^, ^33^, ^37^ However, a few authors have also targeted paraspinal muscles contralateral to the bending side.^30^, ^41^ In CC and ATF, the RA and EAO musclesparticularly when bilaterally hyperactive—are considered the primary contributors to forward trunk flexion, whereas the iliopsoas appears to play only a marginal role.^20^

The efficacy of BTA injections in PD patients with CC is still a topic of debate, as only a few studies have reported favorable postural outcomes.^32^, ^38^, ^40^ Similarly to LTF and PS, polygraphic studies in patients with PD and CC may reveal hyperactivity of the anterior abdominal wall muscles often in association with a dystonic pattern of activation during maximal trunk extension.^11^ It is interesting to note that in the reported cases of CC where no pre‐treatment polygraphy was performed, BTA was ineffective.^34^, ^35^, ^36^ In our opinion, this underscores the importance of identifying hyperactive and dystonic muscles through polygraphy to optimize BTA therapy.

In our study sample we found a statistically significant reduction of the LTF angle values at 1 month follow‐up from BTA injection. These data are consistent with literature data that have claimed postural improvement in PS treated with BTA after injection ipsilateral to bending.^29^, ^31^, ^37^ However, as pointed out by Gandolfi and colleagues, these findings are impinged by extensive variability in injection protocols and BTA dosages across the studies.^42^ We hypothesize that the discrepancy between our findings and prior studies reporting no benefit may be explained by the use of a standardized approach that combines clinical and neurophysiological assessment of APAs. Notably, in our study, 15 patients were treated for LTF, of these 12 (80% of total treated for LTF) showed an improvement in the LTF angle. Moreover, of the 12 patients that showed an LTF angle improvement, 4 (26.7% of total treated for LTF) had an improvement greater than 5°. As far as we know, no validated criteria exist for defining a clinically meaningful response in LTF angle change after BTA.^30^ The 5° cutoff was arbitrarily adopted as clinically significant in previous studies.^30^, ^33^ Although our tool for measuring angles was different from those used in the aforementioned studies, we observed an improvement of LTF angle above this cut‐off in more than one quarter of treated patients. Whether this change has clinical relevance remains uncertain. Instrumental assessment of gait, balance and fall risk assessment could provide additional information on BTA impact on patients’ activity of daily living. However, such assessments were not performed in our study. On the other hand, we did not observe a significant reduction in either the uf‐ATF or lf‐ATF angles 1 month after injection. These findings should be interpreted with caution given the small sample size. Moreover, in ATF, the dystonic drive may, in some patients, coexist with myopathy and spinal structural changes, which can limit the efficacy of chemodenervation. Nevertheless, only 1 patient in our sample could be classified as being in the chronic phase, defined as CC duration longer than 36 months, where myopathic changes are thought to be more likely.^7^, ^8^, ^16^ Notwithstanding the considerable variability across studies, a reduction of the degree of ATF has been observed when the RA and EO muscles were targeted.^42^

Of note, nearly half of the patients reported an improvement in their trunk posture, as assessed by the CGI‐I scale. In particular, 53% of patients treated for PS/LTF and 20% of patients treated for CC/ATF had a CGI‐I scale score for posture inferior to 4. Future studies should investigate whether the perceived improvement in posture correlates with enhanced performance in posture‐targeted physiotherapy.

Another interesting outcome was pain relief measured by a significant reduction in NRS score 1 month after BTA. The reduction of NRS score was seen in 60% of patients treated for PS/LTF as well as in 20% of patients treated for CC/ATF. Notably, 4 patients showed an improvement of NRS score greater than 30% of their initial score. Pain improvement was also reported through the CGI for pain, as 50% patients reported pain improvement. In particular, 60% of patients treated for PS/LTF and 20% of patients treated for CC/ATF had a CGI‐I scale score for pain inferior to 4. We found that angular improvement did not correlate with pain improvement. This suggest that, in addition to postural misalignment, other factors such as disease stage may also contribute to pain onset PD.^1^ Our results are in line with other reports which suggest that BTA injections may reduce axial pain in APAs with either ATF^32^, ^35^, ^40^ or LTF.^30^, ^31^, ^33^, ^37^, ^39^, ^41^

Pain in PD can be classified into nociceptive, neuropathic, or nociplastic type.^50^, ^51^, ^52^ Pain associated with APAs in PD falls within the nociceptive category, since it is caused by mechanical stress on tendons, ligaments, and joint surfaces. Mylius and colleagues found that the most common type in PD patients was nociceptive, followed by nociplastic and, less frequently, neuropathic pain.^53^ In our study group all patients presented nociceptive pain and nobody complained of neuropathic pain, as demonstrated by low score on DN4 questionnaire. Moreover, Al‐Wardat and colleagues found that PS and CC are associated with a higher burden of musculoskeletal, chronic and fluctuation‐related pain in patients with PD.^9^ Thus, pain is a particular concern in PD patients with APAs, and BTA injections may represent a valuable option in chronic back pain treatment. Pain improvement after BTA injections may be attributable to muscle relaxation and/or the consequent reduction of mechanical stress on tendons, ligaments, and joint surfaces resulting from improved posture.

To our knowledge, this is the first study that considers a decision‐making algorithm that combines clinical evaluation with polygraphic electromyography, and concludes with pain assessment before and after BTA treatment. In our opinion, this approach enables a better characterization of the APAs in PD, given that lateral and anterior bending rarely occur in isolation. Some PD patients with APAs may also present with concomitant complex spinal deformities, which can further complicate the management and treatment of these conditions. Future studies should assess the combination of multiple APAs,^3^ as concurrent APAs are associated with greater movement impairment and increased risk of falls.^4^ Furthermore, monitoring changes in bending angles beyond those specifically targeted by BTA injections may help reveal possible connections among different APAs.

The major limitations of this study include the small sample size, the lack of a control group and the restriction to study no more than four pairs of muscles at a time. The short follow‐up is also a limitation, as it does not allow to assess how BTA injections may affect the long‐term evolution of APAs. It should be noted that criteria for detecting a significant change in the angle of LTF or ATF still need to be defined. Therefore, for clinical relevance, instrumental assessment of gait, balance and fall risk should be conducted and, in future studies, correlated with improvements of posture after BTA.

Our study confirms that a clinical and neurophysiological algorithm integrating measures of trunk bending and EMG‐detected muscle hyperactivity can optimize BTA treatment for APAs in PD, improving motor/ non‐motor outcomes. Future studies should evaluate the outcome of synergistic action of BTA and physiotherapy on trunk flexion and pain. Finally, studies with a longer follow‐up are warranted to assess whether BTA injections administered repeatedly over time, may slow the progression of APAs in PD.

## Author Roles

(1) Research project: A. Conception, B. Organization, C. Execution; (2) Statistical analysis: A. Design, B. Execution, C. Review and critique; (3) Manuscript preparation: A. Writing of the first draft, B. Review and critique.

G.A.: 1A, 1B, 1C, 2A, 2B, 3A, 3B

G.S.: 1A, 1B, 1C, 2A, 2B, 3A, 3B

S.C.: 1A, 1B, 1C, 3A, 3B

I.D.V.: 1B, 1C, 3B

M.G.: 1B, 1C, 3B

C.A.A.: 1B, 1C, 3A, 3B

M.T.: 1A, 1B, 1C, 2A, 3A, 3B

C.G.: 1A, 1B, 1C, 3A, 3B

## Disclosures

**Ethical Compliance Statement:** The patients gave written consent to participate in the study. We confirm that all authors have read the Journal's position on issues involved in ethical publication and affirm that this work is consistent with those guidelines. The Verona institutional review board approved the study (CE2399 EMG).

**Funding Sources and Conflicts of Interest:** No specific funding was received for this work. The authors declare that there are no conflicts of interest relevant to this work.

**Financial Disclosures for the Previous 12 Months:** The authors declare that there are no additional disclosures to report.

## Supporting information


**Supplementary Figure S1.** A polygraphic recording of one patient with left lateral trunk flexion; during activation maneuvers. During left trunk flexion we record the physiological activity of the left paraspinal muscles. During right trunk flexion, we record the dystonic hyperactivity of left thoracic paraspinal muscle.

**Supplementary Figure S2.** lf‐ATF, lower fulcrum anterior trunk flexion; LTF, lateral trunk flexion; pre, pre‐treatment; post, post‐treatment; uf‐ATF, upper fulcrum anterior trunk flexion; *, statistically significant reduction of the angle after BTA.

**Supplementary Figure S3.** Legend to the second graph: NRS, numeric rating scale for pain; pre, pre‐treatment; post, post‐treatment; *, statistically significant reduction of the NRS after BTA.

## Data Availability

The data that support the findings of this study are available from the corresponding author upon reasonable request.

## References

[1] Geroin C , Artusi CA , Gandolfi M , Zanolin E , Ceravolo R , Capecci M , et al. Does the degree of trunk bending predict patient disability, motor impairment, falls, and Back pain in Parkinson's disease? Front Neurol 2020;11:207. 32296383 10.3389/fneur.2020.00207 PMC7136533

[2] Doherty KM , Van De Warrenburg BP , Peralta MC , Silveira‐Moriyama L , Azulay JP , Gershanik OS , et al. Postural deformities in Parkinson's disease. Lancet Neurol 2011;10(6):538–549. 21514890 10.1016/S1474-4422(11)70067-9

[3] Cao S , Cui Y , Jin J , Li F , Liu X , Feng T . Prevalence of axial postural abnormalities and their subtypes in Parkinson's disease: a systematic review and meta‐analysis. J Neurol 2023;270(1):139–151. 36098837 10.1007/s00415-022-11354-x

[4] Tinazzi M , Gandolfi M , Ceravolo R , et al. Postural abnormalities in Parkinson's disease: an epidemiological and clinical multicenter study. Mov Disord Clin Pract 2019;6(7):576–585. 31538092 10.1002/mdc3.12810 PMC6749805

[5] Tinazzi M , Geroin C , Bhidayasiri R , et al. Task force consensus on nosology and cut‐off values for axial postural abnormalities in parkinsonism. Mov Disord Clin Pract 2022;9(5):594–603. 35844289 10.1002/mdc3.13460 PMC9274349

[6] Tinazzi M , Geroin C , Gandolfi M , Smania N , Tamburin S , Morgante F , Fasano A . Pisa syndrome in Parkinson's disease: an integrated approach from pathophysiology to management: Pisa syndrome in Parkinson's disease. Mov Disord 2016;31(12):1785–1795. 27779784 10.1002/mds.26829

[7] Artusi CA , Geroin C , Nonnekes J , et al. Predictors and pathophysiology of axial postural abnormalities in parkinsonism: a scoping review. Mov Disord Clin Pract 2023;10(11):1585–1596. 38026508 10.1002/mdc3.13879 PMC10654876

[8] Geroin C , Artusi CA , Nonnekes J , et al. Axial postural abnormalities in parkinsonism: gaps in predictors, pathophysiology, and management. Mov Disord 2023;38(5):732–739. 37081741 10.1002/mds.29377

[9] Al‐Wardat M , Geroin C , Schirinzi T , Etoom M , Tinazzi M , Pisani A , et al. Axial postural abnormalities and pain in Parkinson's disease. J Neural Transm 2023;130(2):77–85. 36550202 10.1007/s00702-022-02576-4

[10] Spuler S , Krug H , Klein C , Medialdea IC , Jakob W , Ebersbach G , et al. Myopathy causing camptocormia in idiopathic Parkinson's disease: a multidisciplinary approach. Mov Disord 2010;25(5):552–559. 20014064 10.1002/mds.22913

[11] Magrinelli F , Geroin C , Squintani G , et al. Upper camptocormia in Parkinson's disease: neurophysiological and imaging findings of both central and peripheral pathophysiological mechanisms. Parkinsonism Relat Disord 2020;71:28–34. 31981996 10.1016/j.parkreldis.2020.01.004

[12] Tinazzi M , Juergenson I , Squintani G , et al. Pisa syndrome in Parkinson's disease: an electrophysiological and imaging study. J Neurol 2013;260(8):2138–2148. 23695587 10.1007/s00415-013-6945-8

[13] Margraf NG , Rohr A , Granert O , Hampel J , Drews A , Deuschl G . MRI of lumbar trunk muscles in patients with Parkinson's disease and camptocormia. J Neurol 2015;262(7):1655–1664. 25929656 10.1007/s00415-015-7726-3

[14] Margraf NG , Wrede A , Rohr A , Schulz‐Schaeffer WJ , Raethjen J , Eymess A , et al. Camptocormia in idiopathic Parkinson's disease: a focal myopathy of the paravertebral muscles. Mov Disord 2010;25(5):542–551. 20108372 10.1002/mds.22780

[15] Wrede A , Margraf NG , Goebel HH , Deuschl G , Schulz‐Schaeffer WJ . Myofibrillar disorganization characterizes myopathy of camptocormia in Parkinson's disease. Acta Neuropathol 2012;123(3):419–432. 22160321 10.1007/s00401-011-0927-7 PMC3282910

[16] Margraf NG , Wrede A , Deuschl G , Schulz‐Schaeffer WJ . Pathophysiological concepts and treatment of camptocormia. J Parkinsons Dis 2016;6(3):485–501. 27314757 10.3233/JPD-160836 PMC5008234

[17] Wolke R , Kuhtz‐Buschbeck JP , Deuschl G , Margraf NG . Insufficiency of trunk extension and impaired control of muscle force in Parkinson's disease with camptocormia. Clin Neurophysiol 2020;131(11):2621–2629. 32932021 10.1016/j.clinph.2020.07.019

[18] Tassorelli C , Furnari A , Buscone S , et al. Pisa syndrome in Parkinson's disease: clinical, electromyographic, and radiological characterization. Mov Disord 2012;27(2):227–235. 21997192 10.1002/mds.23930

[19] Fasano A , Di Matteo A , Vitale C , Squintani G , Ferigo L , Bombieri F , et al. Reversible Pisa syndrome in patients with Parkinson's disease on rasagiline therapy. Mov Disord 2011;26(14):2578–2580. 22170277 10.1002/mds.23918

[20] Furusawa Y , Hanakawa T , Mukai Y , et al. Mechanism of camptocormia in Parkinson's disease analyzed by tilt table‐EMG recording. Parkinsonism Relat Disord 2015;21(7):765–770. 25976984 10.1016/j.parkreldis.2015.02.027

[21] Margraf NG , Rogalski M , Deuschl G , Kuhtz‐Buschbeck JP . Trunk muscle activation pattern in parkinsonian camptocormia as revealed with surface electromyography. Parkinsonism Relat Disord 2017;44:44–50. 28882381 10.1016/j.parkreldis.2017.08.028

[22] Matteo A , Fasano A , Squintani G , et al. Lateral trunk flexion in Parkinson's disease: EMG features disclose two different underlying pathophysiological mechanisms. J Neurol 2011;258(5):740–745. 21079986 10.1007/s00415-010-5822-y

[23] Geroin C . Pisa syndrome in Parkinsons disease: electromyographic quantification of paraspinal and non‐paraspinal muscle activity. Funct Neurol 2017;37(3):143. 10.11138/FNeur/2017.32.3.143 PMC5726350 29042003

[24] Semedo C , Calado A , Dias M , Pedrosa R , Almeida M . Tricks that relieve camptocormia during gait in Parkinson's disease patients. Rev Neurocienc 2001;20(2):236–239.

[25] Gerton BK , Theeler B , Samii A . Backpack treatment for camptocormia. Mov Disord 2010;25(2):247–248. 20077473 10.1002/mds.22909

[26] Barone P , Santangelo G , Amboni M , Pellecchia MT , Vitale C . Pisa syndrome in Parkinson's disease and parkinsonism: clinical features, pathophysiology, and treatment. Lancet Neurol 2016;15(10):1063–1074. 27571158 10.1016/S1474-4422(16)30173-9

[27] Gandolfi M , Tinazzi M , Magrinelli F , et al. Four‐week trunk‐specific exercise program decreases forward trunk flexion in Parkinson's disease: a single‐blinded, randomized controlled trial. Parkinsonism Relat Disord 2019;64:268–274. 31097299 10.1016/j.parkreldis.2019.05.006

[28] Gandolfi M , Geroin C , Imbalzano G , Camozzi S , Menaspà Z , Tinazzi M , Alberto Artusi C . Treatment of axial postural abnormalities in parkinsonism disorders: a systematic review of pharmacological, rehabilitative and surgical interventions. Clin Park Relat Disord 2024;10:100240. 38596537 10.1016/j.prdoa.2024.100240 PMC11002662

[29] Ledda C , Panero E , Dimanico U , Parisi M , Gandolfi M , Tinazzi M , et al. Longitudinal assessment of botulinum toxin treatment for lateral trunk flexion and Pisa syndrome in Parkinson's disease: real‐life, long‐term study. Toxins 2023;15(9):566. 37755992 10.3390/toxins15090566 PMC10536312

[30] Artusi CA , Bortolani S , Merola A , Zibetti M , Busso M , De Mercanti S , et al. Botulinum toxin for Pisa syndrome: an MRI‐, ultrasound‐ and electromyography‐guided pilot study. Parkinsonism Relat Disord 2019;62:231–235. 30442481 10.1016/j.parkreldis.2018.11.003

[31] Matsumoto H , Akahori T , Hatano K , Hashida H . Botulinum toxin treatment of paraspinal muscles for improving abnormal posture in Parkinson's disease. Basal Ganglia 2018;12:1–3.

[32] Todo H , Yamasaki H , Ogawa G , Nishida K , Futamura N , Funakawa I . Injection of Onabotulinum toxin a into the bilateral external oblique muscle attenuated camptocormia: a prospective open‐label study in six patients with Parkinson's disease. Neurol Ther 2018;7(2):365–371. 30094699 10.1007/s40120-018-0108-x PMC6283798

[33] Tassorelli C , De Icco R , Alfonsi E , Bartolo M , Serrao M , Avenali M , et al. Botulinum toxin type A potentiates the effect of neuromotor rehabilitation of Pisa syndrome in Parkinson disease: a placebo controlled study. Parkinsonism Relat Disord 2014;20(11):1140–1144. 25175601 10.1016/j.parkreldis.2014.07.015

[34] Colosimo C , Salvatori FM . Injection of the iliopsoas muscle with botulinum toxin in camptocormia. Mov Disord 2009;24(2):316–317. 10.1002/mds.22249 18973251

[35] Fietzek UM , Schroeteler FE , Ceballos‐Baumann AO . Goal attainment after treatment of parkinsonian camptocormia with botulinum toxin. Mov Disord 2009;24(13):2027–2028. 19645067 10.1002/mds.22676

[36] Von Coelln R , Raible A , Gasser T , Asmus F . Ultrasound‐guided injection of the iliopsoas muscle with botulinum toxin in camptocormia. Mov Disord 2008;23(6):889–892. 18307265 10.1002/mds.21967

[37] Bonanni L , Thomas A , Varanese S , Scorrano V , Onofrj M . Botulinum toxin treatment of lateral axial dystonia in parkinsonism. Mov Disord 2007;22(14):2097–2103. 17685467 10.1002/mds.21694

[38] Azher SN , Jankovic J . Camptocormia: pathogenesis, classification, and response to therapy. Neurology 2005;65(3):355–359. 16087897 10.1212/01.wnl.0000171857.09079.9f

[39] Dupeyron A , Viollet E , Coroian F , Gagnard C , Renard D , Castelnovo G . Botulinum toxin‐A for treatment of Pisa syndrome: a new target muscle. Parkinsonism Relat Disord 2015;21(6):669–670. 25899457 10.1016/j.parkreldis.2015.03.027

[40] Wijemanne S , Jimenez‐Shahed J . Improvement in dystonic camptocormia following botulinum toxin injection to the external oblique muscle. Parkinsonism Relat Disord 2014;20(10):1106–1107. 24981917 10.1016/j.parkreldis.2014.06.002

[41] Santamato A , Ranieri M , Panza F , et al. Botulinum toxin type A and a rehabilitation program in the treatment of Pisa syndrome in Parkinson's disease. J Neurol 2010;257(1):139–141. 19763384 10.1007/s00415-009-5310-4

[42] Gandolfi M , Artusi CA , Imbalzano G , Camozzi S , Crestani M , Lopiano L , et al. Botulinum toxin for axial postural abnormalities in Parkinson's disease: a systematic review. Toxins 2024;16(5):228. 38787080 10.3390/toxins16050228 PMC11125648

[43] Postuma RB , Berg D , Stern M , et al. MDS clinical diagnostic criteria for Parkinson's disease: MDS‐PD clinical diagnostic criteria. Mov Disord 2015;30(12):1591–1601. 26474316 10.1002/mds.26424

[44] Schade S , Mollenhauer B , Trenkwalder C . Levodopa equivalent dose conversion factors: an updated proposal including Opicapone and safinamide. Mov Disord Clin Pract 2020;7(3):343–345. 32258239 10.1002/mdc3.12921 PMC7111582

[45] Finsterer J . EMG‐interference pattern analysis. J Electromyogr Kinesiol 2001;11(4):231–246. 11532594 10.1016/s1050-6411(01)00006-2

[46] Artusi CA , Geroin C , Imbalzano G , et al. Assessment of axial postural abnormalities in parkinsonism: automatic picture analysis software. Mov Disord Clin Pract 2023;10(4):636–645. 37070056 10.1002/mdc3.13692 PMC10105105

[47] Aldegheri S , Artusi CA , Camozzi S , Di Marco R , Geroin C , Imbalzano G , et al. Camera‐ and viewpoint‐agnostic evaluation of axial postural abnormalities in people with Parkinson's disease through augmented human pose estimation. Sensors 2023;23(6):3193. 36991904 10.3390/s23063193 PMC10058715

[48] Artusi CA , Geroin C , Pandino C , et al. Dynamic video assessment of axial postural abnormalities in Parkinson's disease: a pilot study. Mov Disord Clin Pract 2025;12(5):626–637. 39878647 10.1002/mdc3.14329 PMC12070167

[49] Margraf NG , Wolke R , Granert O , et al. Consensus for the measurement of the camptocormia angle in the standing patient. Parkinsonism Relat Disord 2018;52:1–5. 29907329 10.1016/j.parkreldis.2018.06.013

[50] Freynhagen R , Parada HA , Calderon‐Ospina CA , et al. Current understanding of the mixed pain concept: a brief narrative review. Curr Med Res Opin 2019;35(6):1011–1018. 30479161 10.1080/03007995.2018.1552042

[51] Gandolfi M , Geroin C , Antonini A , Smania N , Tinazzi M . Understanding and treating pain syndromes in Parkinson's disease. International Review of Neurobiology [Internet]. Elsevier; 2017 [cited 2025 June 27]:827–858 Available from: https://linkinghub.elsevier.com/retrieve/pii/S0074774217300570. 10.1016/bs.irn.2017.05.013 28805585

[52] Tinazzi M , Gandolfi M , Artusi CA , et al. Advances in diagnosis, classification, and management of pain in Parkinson's disease. Lancet Neurol 2025;24(4):331–347. 40120617 10.1016/S1474-4422(25)00033-X

[53] Mylius V , Perez Lloret S , Cury RG , et al. The Parkinson disease pain classification system: results from an international mechanism‐based classification approach. Pain 2021;162(4):1201–1210. 33044395 10.1097/j.pain.0000000000002107 PMC7977616

