# Supplementary figures and images for: Clinical Algorithm‐Guided Approach to Botulinum Toxin Type A Treatment for Axial Postural Abnormalities in Parkinson's Disease
Source: Mov Disord Clin Pract. 2025 Nov 5;13(4):985–95. doi: 10.1002/mdc3.70408 (PMC13071366; doi:10.1002/mdc3.70408)

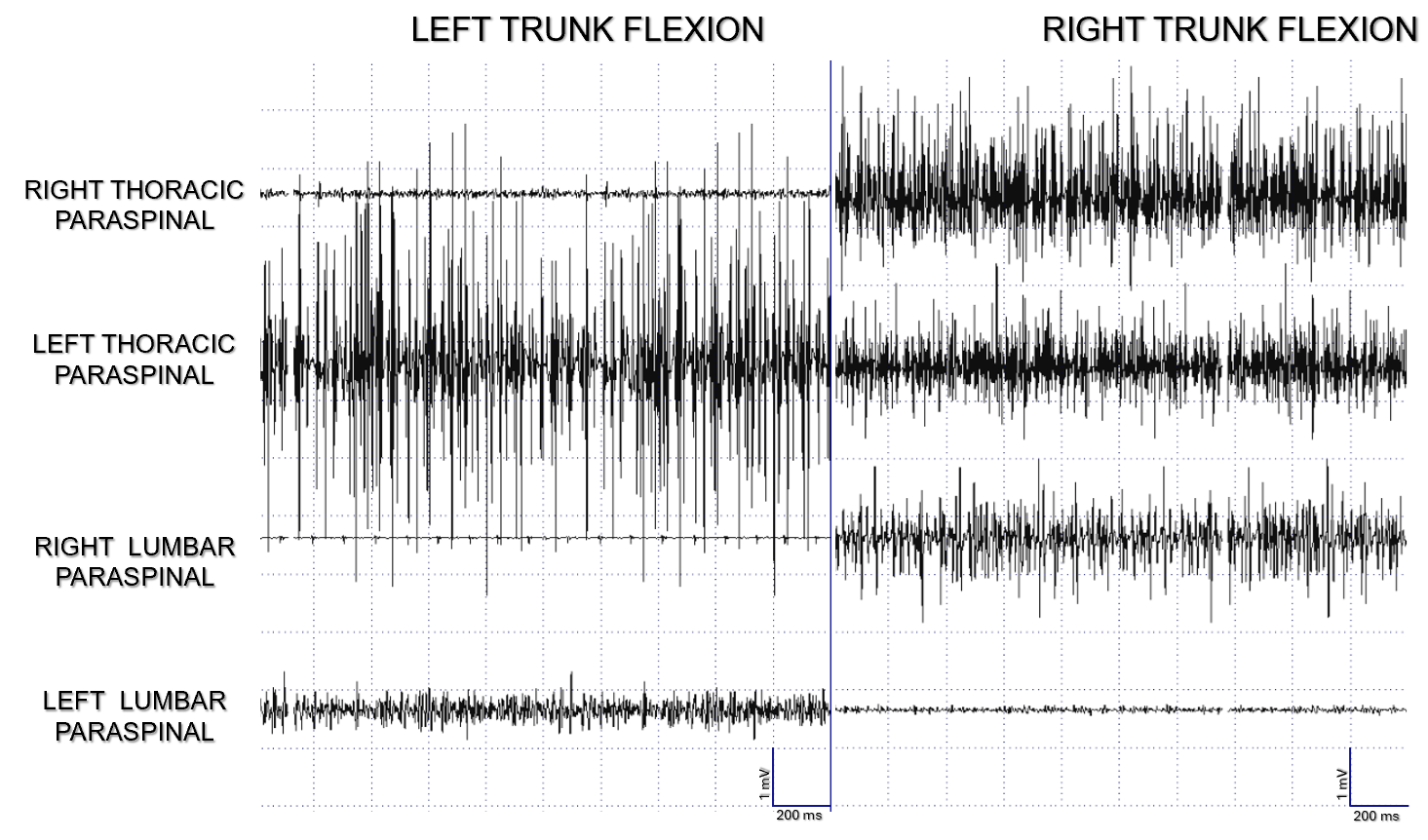

Supplement: Supplementary file 1 — Supplementary Figure S1. A polygraphic recording of one patient with left lateral trunk flexion; during activation maneuvers. During left trunk flexion we record the physiological activity of the left paraspinal muscles. During right trunk flexion, we record the dystonic hyperactivity of left thoracic paraspinal muscle. [file MDC3-13-985-s002.tiff]

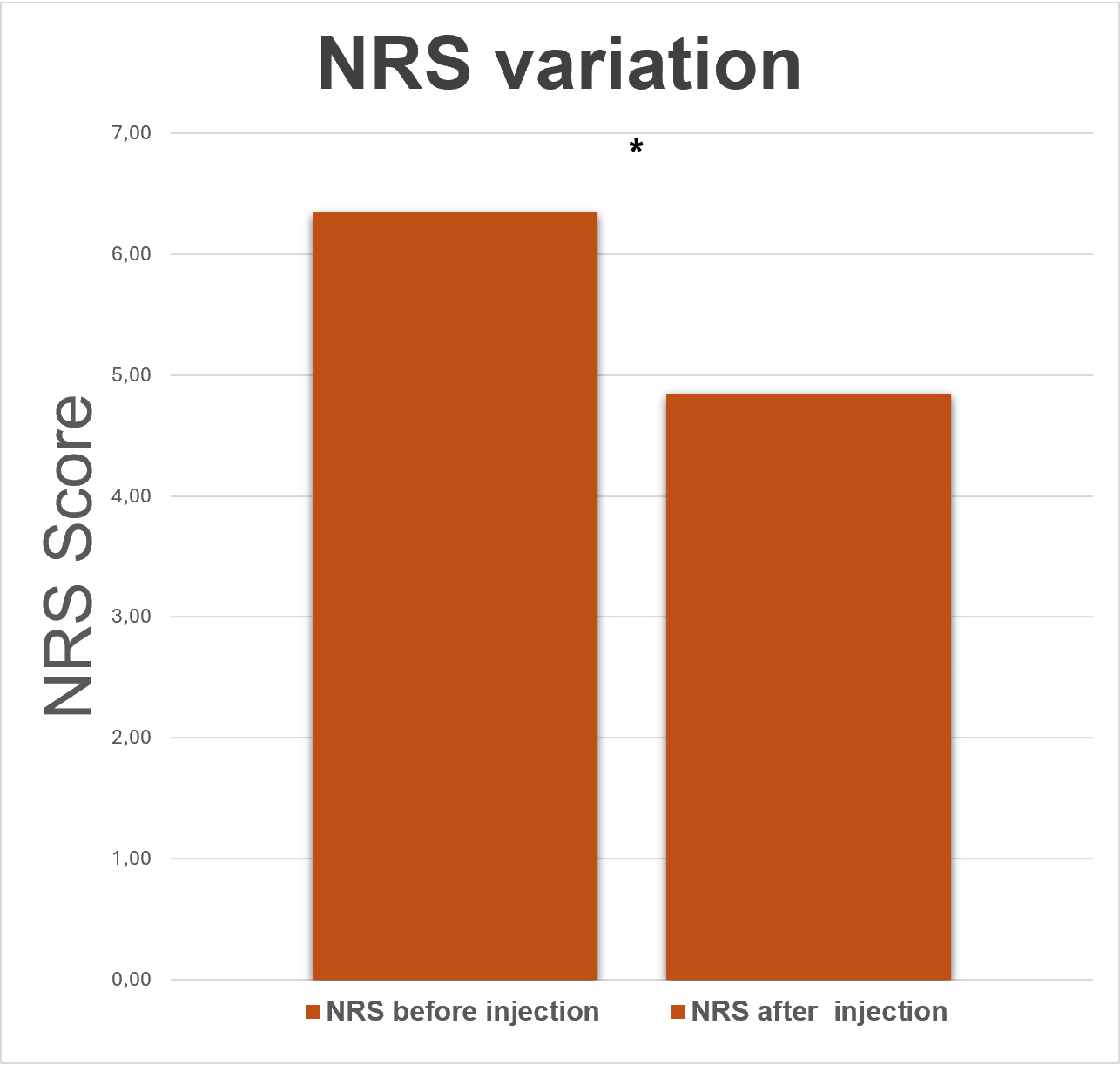

Supplement: Supplementary file 2 — Supplementary Figure S2. lf‐ATF, lower fulcrum anterior trunk flexion; LTF, lateral trunk flexion; pre, pre‐treatment; post, post‐treatment; uf‐ATF, upper fulcrum anterior trunk flexion; *, statistically significant reduction of the angle after BTA. [file MDC3-13-985-s003.tiff]

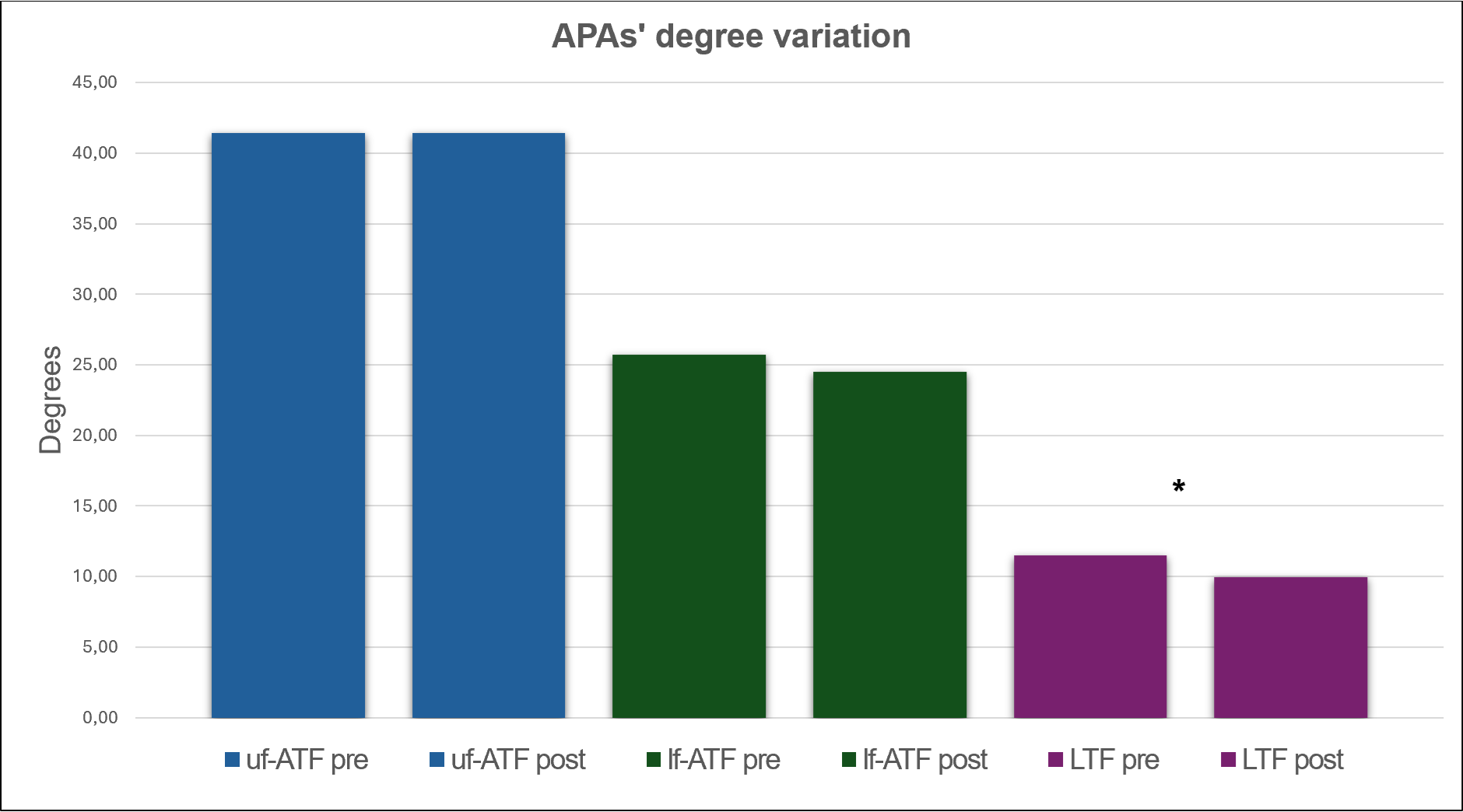

Supplement: Supplementary file 3 — Supplementary Figure S3. Legend to the second graph: NRS, numeric rating scale for pain; pre, pre‐treatment; post, post‐treatment; *, statistically significant reduction of the NRS after BTA. [file MDC3-13-985-s001.tiff]
